# Tobacco-smoking induced GPR15-expressing T cells in blood do not indicate pulmonary damage

**DOI:** 10.1186/s12890-017-0509-0

**Published:** 2017-11-28

**Authors:** Mario Bauer, Beate Fink, Hans-Jürgen Seyfarth, Hubert Wirtz, Armin Frille

**Affiliations:** 1 Department of Environmental Immunology, Helmholtz Centre for Environmental Research-UFZ, Leipzig, Germany; 2 Department of Respiratory Medicine, University of Leipzig, Leipzig, Germany; 3 Leipzig University Medical Center, IFB AdiposityDiseases, Leipzig, Germany

**Keywords:** GPR15, Tobacco-smoking, cg05575921, Biomarker, Lung disease

## Abstract

**Background:**

Recently, it was shown that chronic tobacco smoking evokes specific cellular and molecular changes in white blood cells by an excess of G protein-coupled receptor 15 (GPR15)-expressing T cells as well as a hypomethylation at DNA CpG site cg05575921 in granulocytes. In the present study, we aimed to clarify the general usefulness of these two biomarkers as putative signs of non-cancerous change in homeostasis of the lungs.

**Methods:**

In a clinical cohort consisting of 42 patients with chronic obstructive pulmonary disease (COPD), interstitial lung disease (ILD) and pneumonia and a control cohort of 123 volunteers, the content of GPR15-expressing blood cells as well as the degree of methylation at cg05575921 were analysed by flow-cytometry and pyrosequencing, respectively. Smoking behaviour was estimated by questionnaire and cotinine level in plasma.

**Results:**

Never-smoking patients could be distinguished from former and current smokers by both the proportion of GPR15-expressing T cells as well as cg05575921 methylation in granulocytes, with 100% and 97% specificity and 100% sensitivity, respectively. However, both parameters were not affected by lung diseases. The degrees of both parameters were not changed neither in non-smoking nor smoking patients, compared to appropriate control cohorts of volunteers.

**Conclusions:**

The degree of GPR15-expressing cells among T cells as well as the methylation at cg05575921 in granulocytes in blood are both rather signs of tobacco-smoking induced systemic inflammation because they don’t indicate specifically non-cancerous pathological changes in the lungs.

## Background

Tobacco smoke, worldwide, is one of the major lifestyle inhalative pollutants and can cause severe adverse health effects including the development of chronic obstructive pulmonary disease (COPD) and lung cancer. Although tobacco smoke may cause permanent damage to the lung, it is not clear, which active smoker will actually suffer from smoking-induced adverse health effects. For instance, about 10 to 20% of all heavy cigarette smokers will develop COPD (reviewed in [1]) and only less than 0.5% of tobacco smokers in Germany will develop lung cancer [2–4]. Thus, it is of special interest to determine biomarkers for the prediction of individual health risk over time in the case of tobacco smokers.

Epidemiological studies concordantly revealed an association of active tobacco smoking with single molecular changes of gene expression [5–8] or DNA methylation [8–12] in the whole blood. After taking into account the cell-type’s origin of molecular changes, to date only two meaningful biomarkers for smoking could be described for the tissue blood [13, 14], firstly, the smoking-induced increased frequency of GPR15+ T cells and secondly, the hypomethylation in *AHRR* (aryl-hydrocarbon receptor repressor) gene at cg05575921 in granulocytes.

The GPR15 is a surface membrane-bound G protein-coupled receptor 15 that serves the human immunodeficiency virus type 1 and 2 as a chemokine receptor [15]. It also mediates the homing of lymphocytes to the site of inflammation in the large intestine [16] and of dendritic epidermal T cells to the skin [17]. Although lymphocyte recruitment maintains intestinal immune homeostasis, it also contributes to inflammation [18]. In the mouse, GPR15 is expressed by TH17 and TH1 effector cells and is required for the development of colitis. In contrast, in humans GPR15 is expressed more by pathogenic TH2 cells in ulcerative colitis. Although GPR15-expressing lymphocytes are overrepresented in inflamed tissue of the colon, their frequency in blood remains unchanged in patients with intestinal bowel diseases [19]. Nevertheless, it was recently hypothesized that the up-regulation of *GPR15* gene expression in blood could explain to some extent the health hazards of smoking with regard to chronic inflammatory diseases [20].

The nuclear AHRR serves as a negative feedback modulator by repressing the aryl-hydrocarbon receptor (AHR)-dependent gene expression and may repress inflammatory genes AHR-independently [21]. The AHR is induced by a wide variety of organic endogenous ligands and environmental xenobiotics including compounds of tobacco smoke [22]. The CpG site cg05575921 is located in the gene body of *AHRR*. Its hypomethylation is associated with increased gene expression [13] and thus might promote smoking-induced systemic inflammation through blocking AHR’s suppression of inflammation [23]. In contrast, intestinal inflammation is dampened by AHRR in concert with AHR suggesting a cell-type specific balancing of AHR/AHRR expression in response to microbial, nutritional and other environmental stimuli [24].

The purpose of the present study was to clarify whether these two putative biomarkers in the blood may be early signs of a disturbed homeostasis of the lung and whether they can be used to assess the individual health risk by smoking. Because of the unknown mechanism of smoking-induced cellular changes in blood we analysed these two biomarkers in patients with differently caused non-cancerous lung diseases including (i) COPD, a symptom caused by chronic inflammation in the airways and lung parenchyma and most commonly caused by tobacco smoking, (ii) interstitial lung disease (ILD), indicating pathologic changes in connective tissue, and (iii) pneumonia, an inflammatory disease affecting primarily the alveoli of the lung.

## Methods

### Subjects

For the present study, data from two independent human cohorts were used. One cohort served as a control group comprising of 123 randomly selected pseudonymous blood samples from volunteers obtained from the blood bank at the University of Leipzig (blood donation cohort, details in [13]). Because of the low prevalence of lung diseases [25], this cohort was considered as “healthy”, although volunteers were not examined for lung diseases. Smoking behaviour (yes or no), age and gender were recorded via questionnaires. The second cohort was comprised of 42 patients who were admitted to the hospital and treated for lung diseases, including the acute exacerbation of COPD, ILD and pneumonia, at the Department of Respiratory Medicine at the University Hospital of Leipzig (Table 1).

**Table 1:** Description of the cohorts. In contrast to the lung disease cohort, volunteers were not distinguished between never and former smoker by questionnaire

	Blood donation cohort	Lung disease cohort		
	“Healthy”	COPD	ILD	Pneumonia
Number	123	20	11	11
Age in years, *mean [min-max]*	42.7 [19 - 71]	66.1 [47 - 85]	65.2 [49 - 80]	70.7 [39 -86]
Gender				
Female	56	10	7	1
Male	67	10	4	10
Smoker				
None	91			
Never		1	6	3
Former		11	4	6
Current	32	8	1	2
Anti-inflammatory				
medication				
Prednisolone		8	3	2

*COPD* Chronic obstructive pulmonary disease, *ILD* Interstitial lung disease

The management of patients with COPD was regulated according to the current international guidelines [26, 27]. The severity of airflow limitation was defined by a reduction of forced expired volume in 1 s (FEV_1_) using spirometry, as recommended by the Global Initiative for Chronic Obstructive Lung Disease (GOLD). According to the reduction of the FEV_1_ in patients with FEV_1_/FVC (forced vital capacity <0.7), 4 classes were defined: GOLD 1 (mild): FEV_1_ ≥ 80% predicted, GOLD 2 (moderate): 50% ≤ FEV_1_ < 80% predicted, GOLD 3 (severe): 30% ≤ FEV_1_ < 50% predicted, GOLD 4 (very severe): FEV_1_ < 30% predicted. Twenty patients with COPD were included in this study, of which 16 (80%) suffered from at least severe COPD (GOLD 3-4). Eight of the twenty (40%) patients were admitted due to an acute exacerbation of the COPD and were temporarily treated with anti-inflammatory agents such as systemic glucocorticoids (e.g. prednisolone).

The management of patients with ILD was regulated according to the current international guidelines [28, 29]. Eleven patients with ILD were included, of which 8 (73%) were diagnosed with idiopathic pulmonary fibrosis (IPF). The remaining 3 (27%) non-IPF patients consisted of hypersensitivity pneumonitis, cryptogenic organizing pneumonia (COP), and respiratory bronchiolitis–interstitial lung disease (RB-ILD). All 8 IPF patients were none or former smokers and only two of them were treated with an anti-inflammatory maintenance therapy (prednisolone 2 and 5 mg per day, respectively). One patient with hypersensitivity pneumonitis was treated with an anti-inflammatory combination maintenance therapy (prednisolone and azathioprine 2 and 75 mg per day, respectively).

The management of pneumonia was regulated according to the current international guidelines [30–32]. Eleven patients with pneumonia were included, of which 8 (73%) were diagnosed with a community-acquired pneumonia and 3 (7%) with a hospital-acquired pneumonia.

All participants gave their written informed consent. More detailed smoking behaviour (including pack years, time of cessation) was determined by questionnaire and rechecked by cotinine level in plasma. The study received approval by the Ethics Committee of the University of Leipzig (reference numbers 079-15-09032015 and 199/16-ek).

### Analysis of protein expression at cellular level

GPR15 surface expression concomitant with markers of differentiation on lymphocytes was analysed by flow cytometry. Briefly, 100 μl of blood specimen was incubated with mouse-anti-human-GPR15 antibody (1:500; R&D Systems, Wiesbaden-Nordenstadt, Germany) supplemented with 5% goat serum for 1 h. After washing in PBS/1% fetal calf serum (FCS) the GPR15 was stained with R-phycoerythrin-labelled goat-anti-mouse IgG2b (1:500, 1 h; Biozol, Eching, Germany) following an additional wash step. Thereafter, cells were incubated with 5% mouse serum for 30 min following a double-staining step for leucocyte differentiation markers (1 h; anti-CD3-FITC [Beckman Coulter, Krefeld, Germany], − CD4-BV510, −CD8-PerCP, −CD19-APC-H7 [BD Biosciences, Heidelberg, Germany], −CD16-PerCP, −CD56-PerCP [EXBIO, Prague, Czech Republic]). At the end, erythrocytes were lysed in FACS Lysing solution (BD Bioscience, Heidelberg, Germany) according to manufacturer’s instruction immediately before measurement. All measurements were performed on a FACS Canto II and analysed with the BD FACS DIVA software (version 8.0.1, BD Biosciences, Heidelberg, Germany).

### Analysis of CpG methylation at cg05575921 in granulocytes

Genomic DNA from blood granulocytes separated from PBMC by density gradient centrifugation using Ficoll-Paque (GE Healthcare, Solingen, Germany) was extracted using the Blood DNA extraction kit according to the manufacturer’s protocol (Qiagen, Hilden, Germany). DNA bisulfite treatment was performed using the Epitect kit (Qiagen) according to manufacturer’s instruction. Samples were immediately stored at −20 °C and thereafter simultaneously analysed by pyrosequencing. Methylation assays were designed using the PyroMark Assay Design Software 2.0 (www.qiagen.com). Primer sequences for pyrosequencing are GTGGGGATTGTTTATTTTTGAGAGG (forward), biotin-AACCCTACCAAAACCACTC (reverse) and GGTTTTGGTTTTGTTTTGTA (sequencing). Methylation levels for the CpG site were assessed using Pyromark Q24 pyrosequencer (Qiagen).

### Validation of smoking behaviour - Cotinine ELISA

Smoking behaviour was validated by cotinine measurements in blood. The cotinine concentration was measured in either blood plasma or blood buffy coat samples using the Cotinine direct ELISA Kit according to manufacturer’s instruction (DRG Instruments GmbH, Marburg, Germany).

### Statistical analysis

Discrimination of smoking behaviour into non-, never, former and current smoker was determined by a combination of questionnaire and cotinine level in plasma. Statistical significance of parametric distributed values was calculated with unpaired Student’s t-test. Otherwise, the nonparametric Mann-Whitney U test and Kruskal-Wallis Test were applied for comparison of two or more groups, respectively (Statistica for Windows version 10, [StatSoft Inc. (Europe)]). The linear correlation between single biomarkers and the two independent variables smoking behaviour and lung disease was examined by multiple linear regression. Box plots illustrates median, 25% and 75% percentile, non-outlier max and – min, outlier (circle) and extremes (triangle). All *p*-values <0.05 were considered to be significant. Both sensitivity (proportion of positives that are correctly identified as such) and specificity (proportion of negatives that are correctly identified as such) as statistical measures of a binary classification test were calculated based on a cut-off. The cut-off for GPR15-expression was defined as mean plus two standard deviations of never-smoking patients. The cut-off for cg05575921 methylation was defined as mean minus two standard deviations of never-smoking patients.

## Results

### Use of biomarkers to segregate lung patients by smoking

For the present study, 42 patients with different non-cancerous lung diseases were enrolled (Table 1). This clinical cohort was comprised of 20 patients with chronic obstructive pulmonary disease (COPD), 11 patients with interstitial lung disease (ILD) and 11 patients with pneumonia. Regarding smoking behaviour, the clinical cohort comprised of 10 none, 21 former and 11 current smokers. In contrast to patients with ILD or pneumonia, as expected, patients with COPD were mainly former (55%) or current smokers (40%).

**Fig. 1:**
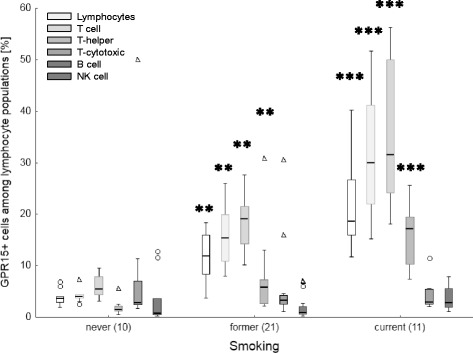
Tobacco smoking-dependent frequency of GPR15+ cells among lymphocyte populations in blood of patients with lung diseases. Frequency of GPR15+ cells are highly specific increased in former and current smokers in T cells. Box plots illustrates median, 25% and 75% percentile, non-outlier max and – min, outlier (circle) and extremes (triangle). Number of patients is enclosed in parenthesis. **, ***; *p*-value of significance <0.01, 0.001 of difference toward never smoker applying Kruskal-Wallis test

**Fig. 2:**
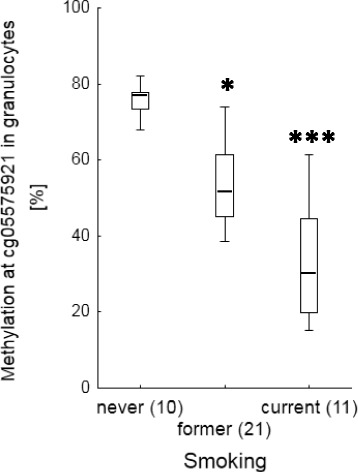
Tobacco smoking-dependent hypomethylation of cg05575921 in granulocytes of blood of patients with lung diseases. Box plots illustrates median, 25% and 75% percentile, non-outlier max and – min. Number of patients is enclosed in parenthesis. *, ***; *p*-value of significance <0.05, 0.001 of difference toward never smoker applying Kruskal-Wallis test

Regardless of the lung disease, the proportion of GPR15+ cells among lymphocyte populations (Fig. 1) as well as the degree of methylation at cg05595721 in granulocytes (Fig. 2) indicated the smoking behaviour with high specificity and sensitivity. The percentage of GPR15+ cells was significantly higher in former and current smokers compared to non-smokers (Table 2 A). These differences were obtained for the whole lymphocyte population, CD3+ T cells, CD4+ T-helper cells, CD8+ T-cytotoxic cells, and CD15/56+ Natural Killer (NK) cells but not in CD19+ B cells. Similar to the percentage of GPR15+ cells, the smoking behaviour was indicated by methylation at cg05595721 in granulocytes. The methylation decreased from 75.9% [confidence interval, 71.9%– 79.9%] in non-smokers to 53.5% [48.5% -58.6%] (*p* = 0.01) and 33.7% [23.9% – 43.5%] (*p* < 0.001) in former and current smokers, respectively. By setting a cut-off for the percentage of GPR15+ cells among CD3+ T cells or CD4+ T-helper cells, a complete discrimination of non-smokers from former/current smokers was obtained (Table 2 B).

**Table 2:** Smoking-dependent proportion of GPR15-expressing cells among different lymphocytes and cg05575921 methylation in granulocytes of blood from patients with lung disease

	GPR15+ cells among blood lymphocytes						CpG methylation
	Lymphocyte	T cell	T-helper	T-cytotoxic	B cell	NK cell	Granulocyte
		[CD3+]	[CD4+]	[CD8+]	[CD19+]	[CD15/56+]	cg05575921
A) Comparison of individuals for smoking							
*mean [confidence interval][%]*							
Smoking							
never [n, 10]	3.9	4.5	5.9	1.9	8.9	3.3	75.9
	[2.8-5.0]	[3.3-5.6]	[4.5-7.4]	[0.9-2.9]	[−1.7-19.4]	[−0.1-6.7]	[71.9-79.9]
former [n, 21]	11.7	16.0	18.5	7	4.9	1.9	53.5
	[9.5-13.8]	[13.3-18.2]	[16.1-20.9]	[4.1-9.8]	[1.9-7.9]	[0.9-2.8]	[48.5-58.6]
current [n, 11]	21.7	31,4	35	16	4	3.6	33.7
	[16.1-27.4]	[24.0-38.9]	[26.3-43.7]	[12.0-20.0]	[2.2-5.9]	[2.0-5.1]	[23.9-43.5]
*p-value* ^*a*^							
Smoking	<0.001	<0.001	<0.001	<0.001	0.953	0.026	<0.001
never vs former	0.004	0.001	0.002	0.006	1.000	1.000	0.01
never vs current	<0.001	<0.001	<0.001	<0.001	1.000	0.132	<0.001
former vs current	0.019	0.010	0.010	0.008	1.000	0.027	0.02
B) Use of cellular parameter as biomarker to predict never smoking status							
cut-off [%]^b^	6.9	7.6	10.0	4.8	38.4	12.9	67.2
Specificity	0.85	1.00	1.00	0.73	0.00	0.00	0.91
Sensitivity	1.00	1.00	1.00	0.90	0.90	1.00	1.00

^a^Kruskal-Wallis-test

^b^mean + 2SD (standard deviation) of never smoker

The administration of anti-inflammatory drug prednisolone did not influenced the proportion of GPR15+ T cells in CD3+ cells among never (U-test, *p* = 0.632), former (*p* = 0.744) or current (*p* = 0.906) smoking patients.

### Biomarkers failed to indicate non-cancerous lung diseases

**Fig. 3:**
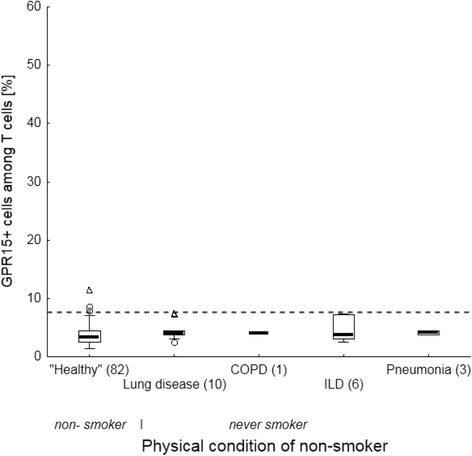
Frequency of GPR15+ cells among T cells in non-smoker of volunteers and never smokers in lung patients. Lung diseases do not influence the frequency of GPR15+ cells in blood The dashed line illustrates the cut-off of 7.6% for discrimination between never and former/current smokers. Box plots illustrates median, 25% and 75% percentile, non-outlier max and – min, outlier (circle) and extremes (triangle). Number of volunteers and patients is enclosed in parenthesis. COPD, chronic obstructive pulmonary disease; ILD, interstitial lung disease

**Fig. 4:**
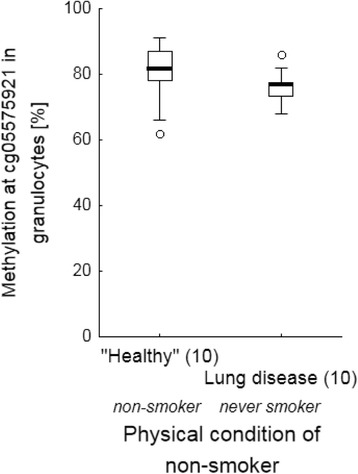
Physical condition-dependent methylation of cg05575921 in granulocytes of blood. Lung diseases do not impair methylation at cg05575921 in granulocytes of blood. Box plots illustrates median, 25% and 75% percentile, non-outlier max and – min outlier (circle). Number of volunteers and patients is enclosed in parenthesis

**Fig. 5:**
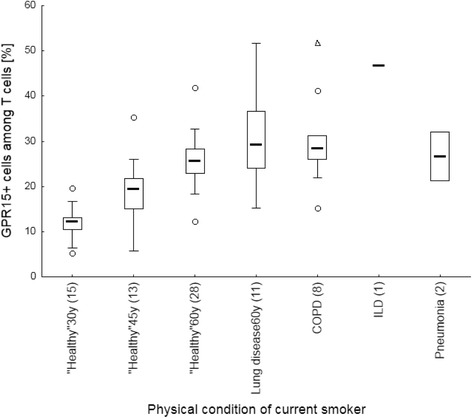
Physical condition-dependent frequency of GPR15+ cells among T cells in current smoker. Lung diseases do not have specific increased, over expected age-matched level, GPR15+ cells among T cells. Box plots illustrates median, 25% and 75% percentile, non-outlier max and – min, outlier (circle) and extremes (triangle). Numbers of age-matched volunteers and patients are enclosed in parenthesis. COPD, chronic obstructive pulmonary disease; ILD, interstitial lung disease; 30y, 45y, 60y, age of 30 ± 5, 45 ± 9 and 60 years, respectively

To distinguish whether blood-derived biomarkers for smoking might indicate a disturbed homeostasis in lung tissue versus being a tobacco smoking-specific sign, their expression was analysed, firstly, in lung patients who never had smoked. As indicated, neither the proportion of GPR15+ T cells (Fig. 3) nor the methylation at cg05597521 in granulocytes (Fig. 4) differed in never-smoked patients with a lung disease compared to non-smoking volunteers. Secondly, current smoking patients did not differ in smoking dosage (pack years)-dependent proportion of GPR15+ T cells from age-matched current smoking volunteers (Fig. 5). Thirdly, despite similar pack years both in former (36.8 [28.7 – 45.0] years) and current smokers (35.8 [27.8 – 44.5] years), the proportion of GPR15+ T cells was decreased (U-test, *p* < 0.001) disease-independently in former-smoking patients (16.0% [13.3% – 18.6%]) compared to current-smoking patients (31.4% [24.4% – 38.0%]). Finally, methylation at cg05595721 in granulocytes of current smoking lung patients (*n* = 11, mean 33.7%) was similar to that of smoking volunteers (*n* = 10, mean 30.0%). Thus, both blood-derived biomarkers did not indicate a disturbed homeostasis in the lung.

## Discussion

From previous observation of gender-independent high variance in proportion of GPR15+ T cells in the blood of current smokers we hypothesized that this biomarker for chronic smoking might suggest a sign of serious adverse health effects of the lung in seemingly “healthy” volunteers [13]. To prove whether biomarkers for chronic smoking may additionally serve as prognostic clinical markers for disturbed homeostasis of the lung, patients with different non-cancerous lung diseases were enrolled in this study. For clinical purposes, we included two biomarkers, the proportion of GPR15+ T cells, a surrogate for the adaptive immunity, along with DNA methylation at the functional cg05597521 site in granulocytes, a surrogate of the innate immunity, because of their high specificity and sensitivity.

In opposite to indicate smoking behaviour of individuals, unexpectedly neither of the biomarker could indicate an expected smoking-induced disturbed homeostasis of the lung in general. None of the never-smoking patients with different lung diseases had shown conspicuous values for at least one of these two biomarkers. Therefore, our assumption that a tobacco smoke-disturbed homeostasis in the lung would activate lung dendritic cells which in terms of migration into regional lymph nodes convey lung-specific information by imprinting T cells via GPR15-expression has not been confirmed. Such T cell imprinting mechanism was well described in mice for effective protection against influenza [33].

To date, the physiological role of GPR15 is not yet clear. An endogenous ligand has actually not been described [34]. In the blood, the expansion of GPR15+ T cells was exclusively described in smokers but not in patients whose GPR15+ cells were found enriched in inflamed tissue such as rheumatic joints [35] or ulcerative colons [19]. Reports in literature on lung tissue are absent, but, the GPR15+ cells do not seem to be enriched in the lungs of smokers. Preliminary data have shown that GPR15+ cells are not enriched in bronchoalveolar lavage from lung patients irrespective of their smoking behaviour (data not shown). It still remains unclear how and why GPR15+ cells in the blood become induced, but it is conceivable that the initial signal does not come from the lung itself. It has been shown that chronic cigarette smokers display a characteristic increase in the number of Langerhans cells (myeloid dendritic cells, mDC) with reduced expression of CCR7 and the lung homing receptor CCR5 [36]. This finding led to the hypothesis that recruitment of mDC into the airways of smokers might reflect the very early reaction of the adaptive immune system to smoke exposure, which shows an increased ability to induce T cell responses but a reduced ability to migrate to draining lymph nodes [37, 38] to initiate differentiation into GPR15+ T cells.

Besides GPR15+ T cells of the adaptive immune system, the innate immune system in form of DNA methylation at cg05597521 in granulocytes is additionally affected by smoking. Segregated into never, former and current smokers both biomarkers did not correlate with each other since the proportion of GPR15+ T cells shows a greater inter-individual variance compared to cg05597521 methylation in granulocytes. However, similar to the proportion of GPR15+ T cells, the methylation at cg05597521 in granulocytes was exclusively impaired by smoking behaviour and not by lung disease in a multiple regression model. This partially agrees with epigenetic analysis of differentially methylated CpG sites in blood. A systematic review did not find any consistent association for lung function or COPD [39]. Furthermore, this site was not found to be smoking-specific in a Korean cohort of COPD patients [40].

Both biomarkers for chronic smoking were included because they are independent of gender and ethnicity. Focusing on patients with lung diseases, it becomes evident that both immunological biomarkers were not influenced by the patient’s use of anti-inflammatory drugs that partially alleviated the immune system. That means that the medication cannot mimic the long-lasting recovery process [8, 12, 41] of smoking-induced adaptation/disturbance of the immune system after cessation. In this study, 8/20 (40%) patients of the COPD cohort were treated with systemic anti-inflammatory agents, such as prednisolone due to an acute exacerbation (usually 0.5 mg per kilogram bodyweight for 3 to 5 days). In the ILD cohort, 3/11 (27%) patients received a maintenance anti-inflammatory therapy of prednisolone (3 to 5 mg per day). Patients treated for pneumonia in this cohort did not receive anti-inflammatory agents.

In contrast to the independency of gender and ethnicity, both biomarkers are dosage-dependent. There is a strong linear correlation of methylation changes in blood with pack years [9]. Based on the association of cell-type specific methylation change at cg19859270, located within the *GPR15* gene body, with the proportion of GPR15+ T cells [14], the expected age-matched proportion of GPR15+ T cells was calculated for volunteers. Compared to the expected value in volunteers, the elder cohort of patients did not show changes in the degree of GPR15+ T cells, indicating further evidence of absent impairment of this biomarker in those lung pathologies.

To date, the physiological role of smoking-affected cells of the adapted (such as T cells) and innate immune system (such as granulocytes) in blood remains elusive. In other tissues, these affected immune cells might participate in chronic inflammatory processes [20]. GPR15 overexpression wass found in granulocytes and monocytes in joints with rheumatoid arthritis (RA) [35] or lymphocytes in inflamed colon [16, 18]. Established RA was additionally accompanied with overexpression of *AHRR* in synovia from patients who smoked. This finding was considered to be responsible for the smoking-affected severity of RA [42]. Similarly, for epithelial cells smoking-induced inflammation in airway was affected by AHRR. In contrast to the proposed role in propagation of inflammation the AHRR may be involved in suppression of inflammation [21]. Therefore, more evidence is needed to interpret appropriately the role of molecular changes in blood by smoking in the context of severity and tissue-specificity of inflammation for an individual risk assessment.

The present study had some limitations. Due to discrimination of patients with different lung diseases by smoking behaviour, there was obtained occasionally an inappropriate number of patients enrolled to perform independent statistical analysis for single lung diseases. For instance, only 1 or 2 patients currently smoked in the ILD or pneumonia group, respectively, and only 1 patient in the COPD group was a never smoker. However, conspicuous biomarker’s values were not found in these patients. With respect to the control group of volunteers, we did not get detailed information about former smoking behaviour because the questionnaire was restricted to only asking if they are a smoker or not. Thus, long-lasting changes in biomarkers of assumed former smoker could influence data for non-smokers in the control group. However, because of their low assumed frequency and the high number of volunteers they seem not to be of statistical importance.

## Conclusion

Evaluating the clinical purpose of recently established high-specific and high-sensitive biomarkers of chronic smoking in blood for prediction of non-cancerous lung diseases, we can conclude that both biomarkers failed to indicate pathological processes in common lung diseases, such as COPD, pulmonary fibrosis, and pneumonia. Therefore, the induction of an excess of GPR15+ T cells in blood remains restricted to chronic tobacco consumption. Whether GPR15 represents a target for therapeutic interventions of tissue inflammation remains elusive since the general physiological role of the immunocompetent GPR15+ cells has not been determined to date.
